# Seasonal environmental fluctuations alter the transcriptome dynamics of oocytes and granulosa cells in beef cows

**DOI:** 10.1186/s13048-024-01530-0

**Published:** 2024-10-14

**Authors:** Kamryn Joyce, Ahmed Gad, Nico G. Menjivar, Samuel Gebremedhn, Daniella Heredia, Georgia Dubeux, Maria Camila Lopez-Duarte, Joao Bittar, Angela Gonella-Diaza, Dawit Tesfaye

**Affiliations:** 1 North Florida Research and Education Center, University of Florida, Marianna, FL 32446 USA; 2 Animal Reproduction and Biotechnology Laboratory, Department of Biomedical Sciences, Colorado State University, Fort Collins, CO 80523 USA; 3 Department of Animal Production, Faculty of Agriculture, Cairo University, Giza, 12613 Egypt; 4 J.R. Simplot Company, 1099 W. Front St, Boise, ID 83702 USA; 5 Large Animal Clinical Sciences, College of Veterinary Medicine, University of Florida, Gainesville, FL 32610 USA

**Keywords:** Oocytes, Granulosa cells, Heat stress, Seasonality, Transcriptome, Beef cows

## Abstract

**Background:**

Examining the mechanistic cellular responses to heat stress could aid in addressing the increasing prevalence of decreased fertility due to elevated ambient temperatures. Here, we aimed to study the differential responses of oocytes and granulosa cells to thermal fluctuations due to seasonal differences. Dry beef cows (*n* = 10) were housed together, synchronized and subjected to a stimulation protocol to induce follicular growth before ovum pick-up (OPU). Two OPU’s were conducted (summer and winter) to collect cumulus-oocyte-complexes (COCs) and granulosa cells. In addition, rectal temperatures and circulating blood samples were collected during OPU. Oocytes were separated from the adherent cumulus cells, and granulosa cells were isolated from the collected OPU fluid. RNA was extracted from pools of oocytes and granulosa cells, followed by library preparation and RNA-sequencing. Blood samples were further processed for the isolation of plasma and leukocytes. The transcript abundance of *HSP70* and *HSP90* in leukocytes was evaluated using RT-qPCR, and plasma cortisol levels were evaluated by immunoassay. Environmental data were collected daily for three weeks before each OPU session. Data were analyzed using MIXED, Glimmix or GENMOD procedures of SAS, according to each variable distribution.

**Results:**

Air temperatures (27.5 °C vs. 11.5 °C), average max air temperatures (33.7 °C vs. 16.9 °C), and temperature-humidity indexes, THI (79.16 vs. 53.39) were shown to contrast significantly comparing both the summer and winter seasons, respectively. Rectal temperatures (Summer: 39.2 ± 0.2 °C; Winter: 38.8 ± 0.2 °C) and leukocyte *HSP70* transcript abundance (Summer: 4.18 ± 0.47 arbitrary units; Winter: 2.69 ± 0.66 arbitrary units) were shown to increase in the summer compared to the winter. No visual differences persisted in *HSP90* transcript abundance in leukocytes and plasma cortisol concentrations during seasonal changes. Additionally, during the summer, 446 and 940 transcripts were up and downregulated in oocytes, while 1083 and 1126 transcripts were up and downregulated in the corresponding granulosa cells, respectively (Fold Change ≤ -2 or ≥ 2 and FDR ≤ 0.05). Downregulated transcripts in the oocytes were found to be involved in ECM-receptor interaction and focal adhesion pathways, while the upregulated transcripts were involved in protein digestion and absorption, ABC transporters, and oocyte meiosis pathways. Downregulated transcripts in the granulosa cells were shown to be involved in cell adhesion molecules, chemokine signaling, and cytokine-cytokine receptor interaction pathways, while those upregulated transcripts were involved in protein processing and metabolic pathways.

**Conclusion:**

In conclusion, seasonal changes dramatically alter the gene expression profiles of oocytes and granulosa cells in beef cows, which may in part explain the seasonal discrepancies in pregnancy success rates during diverging climatic weather conditions.

**Supplementary Information:**

The online version contains supplementary material available at 10.1186/s13048-024-01530-0.

## Background

With the rise of climate change becoming more prevalent over the years, it has been postulated that the duration and severity of heat stress will continually increase, posing detrimental impacts to the livestock industry, notably the beef and dairy sectors [1]. Excessive heat loads on animals negatively impact animal welfare and performance, with evident reductions in reproductive efficiencies, as one of the leading heat stress-related impacts affecting livestock productivity [2]. Additionally, it is well-documented that heat stress negatively impacts the reproductive performance of dairy and beef cattle through alterations in follicular development, oocyte maturation, and granulosa cells’ function [3, 4]. Therefore, understanding the molecular changes in oocytes and follicular cells associated with environmental heat stress in beef cows could provide a unique opportunity to identify molecular targets for future managerial interventions and the development of therapeutic strategies.

Seasonal studies in cattle have shown that elevated temperatures compromise oocyte development [5]. Rocha et al. [6] observed the effects of environmental temperatures and humidity on the quality and developmental capacity of bovine oocytes from Holstein cows [6]. Results from this study showed that a higher percentage of morphologically normal oocytes were produced during the cooler season than during the warmer season, while the percentage of fertilized oocytes that developed to the 2-cell, 8-cell, and morula stages, were also greater during the cooler season. Moreover, previous studies have duly indicated that altered oocyte developmental competence from direct exposure to heat stress also negatively hampers their mitochondrial function by impacting ATP levels and redox regulation [7].Furthermore,, heat stress also has negative impacts on the function of granulosa cells. Disturbances in granulosa cells’ quality and impairments to their proliferation capacity may indirectly affect follicular development and oocyte maturation, resulting in impaired embryo development and subsequent failures in pregnancy establishment [4]. Furthermore, in mice, it was shown that the exposure to heat stress increased the proportion of granulosa cells undergoing apoptosis and the number of atretic follicles, while reducing the intrafollicular aromatase activity, as well as circulating estradiol concentrations [8]. Additional studies are required to examine the molecular mechanisms underlying the response of oocytes and granulosa cells to heat stress, specifically in beef cattle.

A recent study from our group [9] investigated the encapsulated microRNA cargo of extracellular vesicles (EVs) derived from the follicular fluid of beef cows in response to seasonal environmental changes. In that study, several miRNAs were differentially expressed in EVs derived from the summer versus the winter, with precise candidates like miR-10a, miR-10b, miR-184, miR-19b, and miR-452 upregulated and miR-1246, miR-199b, and miR-370 downregulated during summer compared to the winter groups. Interestingly, those DE-miRNAs were found to be involved in the regulation of signaling pathways including WNT-, p53-, mTOR-, Hippo, and FoxO-signaling pathways, which are the dominant regulatory pathways involved in mammalian follicular development. As follicular fluid EV molecular cargos are direct determinants and a reflection of the physiology of the cells amidst the follicular microenvironment including cumulus cells, granulosa cells, and theca cells, the results of our previous study led us to hypothesize that the oocytes and the surrounding granulosa cells respond to environmental seasonal fluctuations in heat stress via altering their transcriptome profile. Therefore, in the present study, we aimed to investigate the transcriptome dynamics of GV-stage oocytes and granulosa cells, determining their response to fluctuating environmental temperatures during summer and winter seasons.

## Materials and methods

### Animals

This experiment was conducted at the UF/IFAS North Florida Research and Education Center in Marianna, FL, and was approved by the Institutional Animal Care and Use Committee (IACUC ID: IACUC202200000706). Eleven non-pregnant and non-lactating, multiparous *Bos taurus* cows crossbreed were clinically examined via transrectal ultrasound to evaluate their reproductive tract and cyclicity status. Only animals without clinical abnormalities showing at least one corpus luteum were enrolled in the study. Cows remained together for the duration of the experiment (February-August) and were maintained in outdoor pens with Bahia grass and fed bermudagrass hay to meet the nutritional requirements of mature cows with ad libitum access to water and mineralized salt during the duration of the study. One cow died before the second OPU session and therefore, was completely removed from the study.

### Synchronization, follicular stimulation, and ovum pick-up

**Fig. 1:**
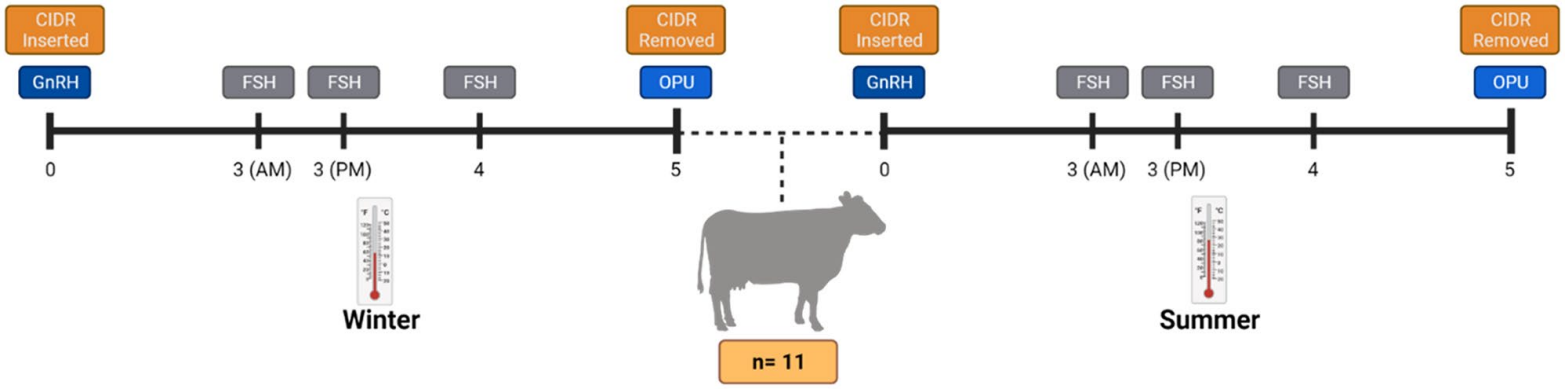
Hormonal protocol used in the present study. Briefly, a 5-day CIDR and FSH synchronization and stimulation protocol was used to induce follicular growth before each OPU session (Winter and Summer). Created with BioRender.com

Cows were subjected to a synchronization and FSH-based stimulation protocol to induce follicular growth as indicated in the hormonal experimental design outlined in Fig. 1. On day 0, an intravaginal P4 device (Eazi-Breed™ CIDR^®^; 1.38 gr P4; Zoetis Animal Health; Kalamazoo, MI, USA) was inserted and an intramuscular injection of a GnRH analog (100 µg of synthetic gonadorelin hydrochloride; Factrel; Zoetis Animal Health; Kalamazoo, MI, USA) was administrated. Follicular growth was stimulated by administering exogenous FSH (porcine pituitary-derived follicle-stimulating hormone; Folltropin^®^; Vetoquinol; Canada). Briefly, on day 3, 105 IU of FSH were administrated intramuscularly twice, once in the morning (7 am) and once in the afternoon (6 pm). Next, on day 4, one injection of 70 IU of FSH was administered in the morning (7 am). Finally, the CIDR was removed on day 5 (morning), and rectal temperature was collected. Transrectal ultrasounds were then conducted to assess the number of antral follicles and the presence of a CL. OPU was performed as previously reported [9] using a real-time B-mode ultrasound scanner (Mindray 2200; Mindray Bio-Medical Electronics, Shenzhen, China) equipped with a 5-MHz micro-convex transducer (Mindray model 65C15EAV, Mindray Bio-Medical Electronics, Shenzhen, China) and coupled to a follicular aspiration guide (WTA, São Paulo, Brazil) and a stainless-steel guide. The follicular puncture was performed using a disposable 18 G hypodermic needle connected to a 50-mL conical tube via a suitable silicon tubing system (WTA). The pressure for aspiration was maintained using a vacuum pump (WTA model BV-003, WTA) with negative pressure adjusted between 60 and 80 mmHg. Only follicles from ~ 4 mm to ≤ 8 mm were aspirated. Follicles ≥ to 9 mm were starting a dominance phase and were not aspirated. After the OPU of both ovaries, the aspiration system was replaced with a new one before conducting OPU for the next cow. OPUs from winter and summer were conducted in February and August, respectively by the same operator.

### Sample collections

#### Collection of cumulus-oocyte-complexes and oocyte isolation

Following OPU procedures for each animal, COCs were allowed to settle to the bottom of the collection tube. The uppermost aqueous fluid above the pelleted aspirate was then collected with a pipette and transferred to a new tube. Following searching the dense collection of cellular debris and tissue remnants, COCs were washed three times with holding media (ABT complete flush; ABT 360, LLC; Pullman, WA, USA). Grading of COCs was performed based on the criteria set by the International Embryo Technology Society [10]. Grade 1: >5 layers of even cumulus cells with even cytoplasm; Grade 2: three to five layers of cumulus cells, mostly even distributed, and even cytoplasm; Grade 3: < 3 layers of dense compact cumulus cells, often uneven, and abnormally small oocytes with clear, granular, and uneven cytoplasm; Grade 4: degenerated. Grade 3 and 4 COCs were discarded and not included in further analyses of this study.

Collected grades 1 and 2 COCs were immediately subjected to enzymatic and mechanical efforts to remove adhered cumulus cells using 10,000 units/mL hyaluronidase (Sigma H-4272) and a high-speed vortex. One ml of holding medium was added to each tube, and the contents were transferred to a separate petri dish. Denuded oocytes (germinal vesicle stage, GV) were then isolated and washed twice in PBS. Every oocyte was closely checked for the presence of somatic cells under a stereomicroscope. Finally, the oocytes were transferred individually to a new tube with minimal volume of PBS and immediately snap-frozen in liquid nitrogen. All samples were stored at -80^o^C until further analysis.

#### Collection of granulosa cells

For individual animals, the uppermost aqueous phase of the OPU collection fluid was transferred to a new 15 ml falcon tube and centrifuged at 750 ×g for 5 min to pelletize the cells. Granulosa cells were then immediately snap-frozen and stored at -80^o^C until further use.

#### Collection of plasma samples

At the time of each OPU, blood samples were collected using EDTA blood collection tubes (K2 EDTA BD Vacutainer^®^; Ref. 366643; BD Vacutainer; United Kingdom). OPU session happened in the morning period for both, summer and winter collections. Collection tubes containing the blood samples were centrifuged at 3000 ×g for 15 min to separate the plasma from the blood cells. The plasma was pipetted into microtubes and stored at -80^o^C for downstream analysis.

#### Leukocytes isolation

The white blood cell layer resulting from harvesting the plasma was then used to harvest the buffy coat. Briefly, the white layer was transferred into a 15 ml falcon tube using a sterile pipette and diluted in 3x the volume (~ 6 ml) of distilled water. Cells were mechanically suspended via pipetting, followed by centrifugation at 500×g for 15 min at 18^o^C. The supernatant was removed, and the pellets were scraped against a rack to unstick the cells. This step was repeated until the pellet was cleared of all red blood cells (2–3 times). Finally, the resuspended cells were transferred to a 2 ml microtube and centrifuged at 4000 ×g for 10 min. Without disturbing the cell pellet, the supernatant was removed, and the ensuing leukocytes were then immediately snap-frozen and stored at -80^o^C for downstream application.

### Climatic conditions

Data for climatic conditions were collected daily for three weeks before each OPU session using the Florida Automated Weather Network (FAWN) [11]. The following weather measurements were taken from FAWN: temperature (average and maximum) and relative humidity. The temperature humidity index (THI) was calculated using the following equation: THI = (1.8 × T + 32) - [(0.55–0.0055 × RH) × (1.8 × T − 26)]. Where T = air temperature (°C) and RH = relative humidity (%) [12].

### Plasma cortisol assay

Circulating plasma cortisol concentrations were quantified using the automated IMMULITE^®^ 2000 Xpi cortisol Immunoassay kit (Cat. No. L2KCO2, Siemens Healthcare, CA, USA). Briefly, per the manufacturer’s recommendations, the cortisol kit was adjusted for each lot number with a calibration range of 10 to 500 ng/mL. Commercially available tri-level (26.8, 149, and 254 ng/mL) quality control samples (Lyphocheck Immunoassay Plus Control, Cat. No. 370, Bio-Rad, Hercules, CA, USA) were run daily before the experimental samples. Quality control samples were considered to pass if the coefficient of variation (CV) was below 10% for each level and the replicates were within the manufacturer’s reported range. Before analysis, samples were thawed at room temperature and run in duplicates. An internal calculation of quantification was reported for each duplicate, and these values were averaged to determine the CV. All concentrations reported have a CV of less than 10%.

### Total RNA extraction, reverse transcription, and quantitative real-time PCR of leukocytes

Total RNA was extracted from leukocytes using the PureLink™ RNA Mini Kit (Cat. No. 12183025; Thermo Fisher Scientific™; Austin, TX, USA) according to the manufacturer’s instruction. Samples were homogenized in 600 µL of lysis buffer containing 1% mercaptoethanol. The “On-Column PureLink^®^ DNase Treatment Protocol” from the PureLink™ DNase Set (Cat. No. 12185010; Thermo Fisher Scientific™) was performed on individual samples to remove DNA contaminants. RNA yield and purity were analyzed using the NanoDrop™ One Spectrophotometer (Thermo Fisher Scientific™) with criteria set that the yield (> 20 ng/µL) and the purity (absorbance 260/280 ratio ~ 2). Cows containing valid samples from summer and winter, were keep for further analysis. Samples were then stored at -80 °C until further use.

RNA samples (*n* = 7 in summer and *n* = 7 in winter) were reverse transcribed to cDNA using the High-Capacity cDNA Reverse Transcription Kit (Cat. No. 4368814; Thermo Fisher Scientific™) following the manufacturer’s instruction. Briefly, 500 ng of RNA and ensuing reagents were combined and subjected to incubation in a Bio-Rad C1000 Touch™ Thermal Cycler at an initial temperature of 25^o^C for 10 min, followed by 37^o^C for 120 min, 85^o^C for 5 min, and then 4^o^C. The final output volume of 20 µL of cDNA was then diluted 1:80 with DEPC-treated water and stored at -20 °C for further qRT-PCR reactions.

Primers used for quantitative real-time PCR (qPCR) are indicated in Supplementary Table 1. The characteristics of the primers were checked in The OligoAnalyzer™ Tool software, (Integrated DNA Technologies, Biodynamics, https://www.idtdna.com/pages/tools/oligoanalyzer?returnurl=%2Fcalc%2Fanalyzer), while the specificity was compared by BLAST1 (NCBI, http://blast.ncbi.nlm.nih.gov). each primer pair were previously validated to establish primer pair efficiency (standard curves) and optimal primer concentration (concentration test). For all reactions (target and reference genes), a master mix containing SYBR Green (Cat. No. 1725274; SsoAdvanced Universal SYBR Green Supermix; Bio-Rad), forward and reverse primers, and DEPC-treated water were prepared in a 2 ml microtube. qPCR reactions were conducted in triplicates and prepared in a 96-well PCR plate (Cat. No. HSP9601; Bio-Rad) containing 16 µL of the master mix and 4 µL of the diluted cDNA samples. No template controls (NTC) without cDNA were run in each PCR plate. The Bio-Rad CFX software was used to run the protocol as follows: denaturation at 95^o^C for 3 min, followed by 40 cycles of 95^o^C for 10 s, and 60^o^C for 30 s, and finally, annealing/extension and plate read at 65^o^C for 5 s and then melting curve analysis at 95^o^C + 0.5^o^C. No amplification was detected in NTC. RefFinder [13, 14] was used to determine the stability of the reference genes, and according to their Cq, *ACT-B*, and *RPL15* were the most stable reference genes. Analysis to determine the relative expression of the genes of interest was done using the PFAFFL equation [15], considering each primer’s efficiency and following normalization using the geometric mean of the selected reference genes (*ACT-B* and *RPL15*). qPCR products from reactions containing target and reference genes primers were submitted to agarose gel electrophoresis and sequencing and identities were confirmed.

### Preparation of RNA from oocytes and granulosa cells for sequencing

Total RNA extraction of oocytes was performed after pooling 2–4 oocytes from a single cow and using the Arcturus™ PicoPure™ RNA (Thermo Fisher Scientific™) according to the manufacturer’s instruction Total RNA from granulosa cells was extracted using the miRNeasy mini kit (Qiagen, Hilden, Germany). For both types of samples (oocytes and granuloma cells), on-column DNA digestion was performed using the RNase-Free DNase Set (Qiagen) to remove genomic DNA contaminants. The concentration and integrity of the isolated samples were then assessed with NanoDrop 8000 spectrophotometer (Thermo Fisher Scientific™) and Agilent 2100 Bioanalyzer (Agilent Technologies, CA), respectively. RNA samples were pooled from two individual animals to create five biological replicates (*n* = 2 cows/pool, 5 pools per season) for both oocytes and granulosa cells, collected during the summer and winter. RNA samples were stored at -80 °C until sequencing.

### Library preparation and RNA-sequencing

For library preparation, messenger RNA was purified from total RNA using poly-T oligo-attached magnetic beads. After fragmentation, the first strand of cDNA was synthesized using random hexamer primers, followed by the second strand of cDNA synthesis. The library was ready after the end repair, A-tailing, adapter ligation, size selection, amplification, and purification. The libraries were checked with a Qubit DNA HS Assay Kit in a Qubit 2.0 Fluorometer (Thermo Fisher Scientific™) for concentration, qPCR for quantification, and a bioanalyzer (Agilent Technologies) for the size distribution. Quantified libraries were pooled and sequenced using a NovaSeq6000 sequencing instrument (Illumina, Inc., San Diego, CA, USA) as paired end reads (150 bases).

### Sequencing data analysis

FASTQ files were generated for each sample using the software bcl2fastq (Illumina Inc., San Diego, CA), and their quality was checked using the FastQC tool version 0.11.9. Data were analyzed using the CLC Genomics Workbench, version 21 (Qiagen). Raw sequencing reads were trimmed based on quality score (Q-score > 30), ambiguous nucleotides (maximum two nucleotides allowed), read length (≥ 15 nucleotides), and after removing adapter sequences. One replicate from oocyte/summer group didn’t pass the quality control check. Therefore, we decided to exclude this replicate and its corresponding oocyte/winter and granulosa cell replicates from further analysis. Reads were mapped to the bovine reference genome (ARS-UCD1.3) applying the default software parameters. Data were normalized using the trimmed mean of the M-values normalization method (TMM normalization) [16] and presented as transcripts per million (TPM). The expression threshold was determined by the zFPKM method using the zFPKM R package v.1.16.0 [17]. Genes with zFPKM > − 3 in all replicates were considered expressed. Differential expression analysis was done using the Differential Expression tool based on a negative binomial Generalized Linear Model (GLM) function. Differentially expressed genes (DEGs) were filtered based on fold change (FC ≥ 2) and the P-adjusted value (FDR < 0.05) [18]. The raw FASTQ files and processed CSV files have been deposited into NCBI’s Gene Expression Omnibus (GEO) with the accession numbers (GSE235170, GSE235171) for the oocytes and granulosa cells, respectively.

After the identification of the DEGs in each cell type separately, a comparative analysis has been conducted to elucidate the unique and shared molecular responses of oocytes and granulosa cells to elevated environmental temperatures. Specifically, we focused on genes that were commonly dysregulated in both cell types during the summer followed by pathway enrichment analysis to identify the biological pathways impacted by these DEGs.

### Pathways and ontological classification analysis

The DEGs were submitted to the Database for Annotation, Visualization, and Integrated Discovery (DAVID) Bioinformatics web tool v. 2021 [19] for pathways and ontological classification enrichment analysis. Pathways and biological processes (BP) were determined from the KEGG database [20] and GOTERM_BP_DIRECT annotation set, respectively.

### Statistical analysis

Rectal temperature, plasma cortisol concentration, and HSP70 and HSP90 transcript abundance in leukocytes were analyzed using the MIXED procedure of SAS version 9.3 (SAS/STAT^®^, SAS Inst. Inc., Cary, NC) with cow as a random effect. The number of follicles and oocytes were analyzed by negative binomial regression analyses using the GENMOD procedure. The proportion of grade 1, 2, 3, and 4 oocytes were analyzed by ANOVA using the GLIMMIX procedure with logit function. Square-root transformation was applied when residuals were not normally distributed according to the Shapiro-Wilk and Kolmogorov-Smirnov tests. For all models, cow was considered the experimental unit, and data are presented as mean ± standard error of the mean. We set the significance level for rejecting the null hypothesis to *P* ≤ 0.05 and considered trends significant at 0.05 ≤ *P* ≤ 0.10.

## Results

### Climatic conditions

**Fig. 2:**
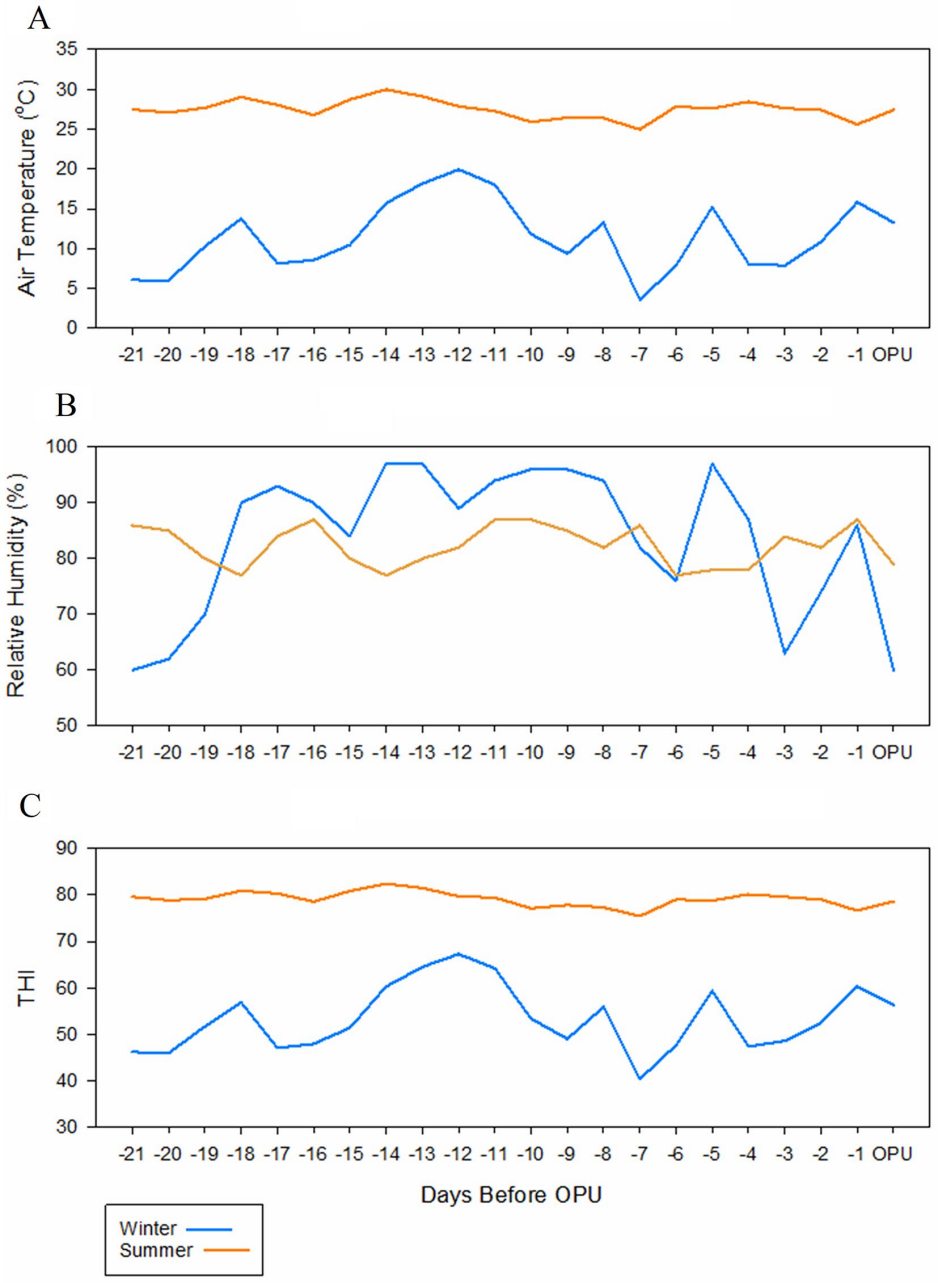
Climatic conditions were collected for twenty-one days before each OPU session. **A**) Average air temperature, **B**) Relative humidity, **C**) Temperature humidity index (THI). Data were collected using the Florida Automated Weather Network (FAWN; https://fawn.ifas.ufl.edu/data/reports/)

As expected, the average air temperature (27.5 °C vs. 11.5 °C), average maximum air temperature (33.7 °C vs. 16.9 °C), and relative humidity (82.3% vs. 83.5%) during the summer and winter, respectively, differed considerably (Fig. 2). After using the equation to calculate the THI for the 3 weeks prior to each sample collection, the summer estimate was 79.2, while the winter was 53.4 (Fig. 2; Table 1). Accordingly, the Livestock Safety Weather Index [21], a common resource used to classify the intensity of the THI values, recognizes four primary categories: normal, alert, danger, and emergency. Based on this criterion, over the three weeks preceding the OPU collections, the winter had 100.0% normal THI days, while the summer had 59.1% of the days considered as ‘alert’ and 40.9% considered as ‘danger’ THI thresholds (Table 1).

**Table 1:** Descriptive statistics for the temperature humidity index (THI) average values for 21 days before each OPU session

Season	Mean	SD	Min	Max	CV, %	% Days THI normal	% Days THI alert	% Days THI danger
Winter	53.4	7.1	40.4	67.3	13.3	100.00	0	0
Summer	79.2	1.6	75.5	82.4	2.0	0	59.1	40.9

SD, Standard deviation; Min, Minimum; Max, Maximum; CV, Coefficient of variation

Thresholds cutoff points from *Livestock Weather Safety Index (LCI*,* 1970)*: THI ≤ 74 = Normal, 74 > THI ≤ 79 = Alert, 79 > THI ≤ 84 = Danger, and THI 84 = Emergency

### Rectal temperature and plasma cortisol concentration

**Fig. 3:**
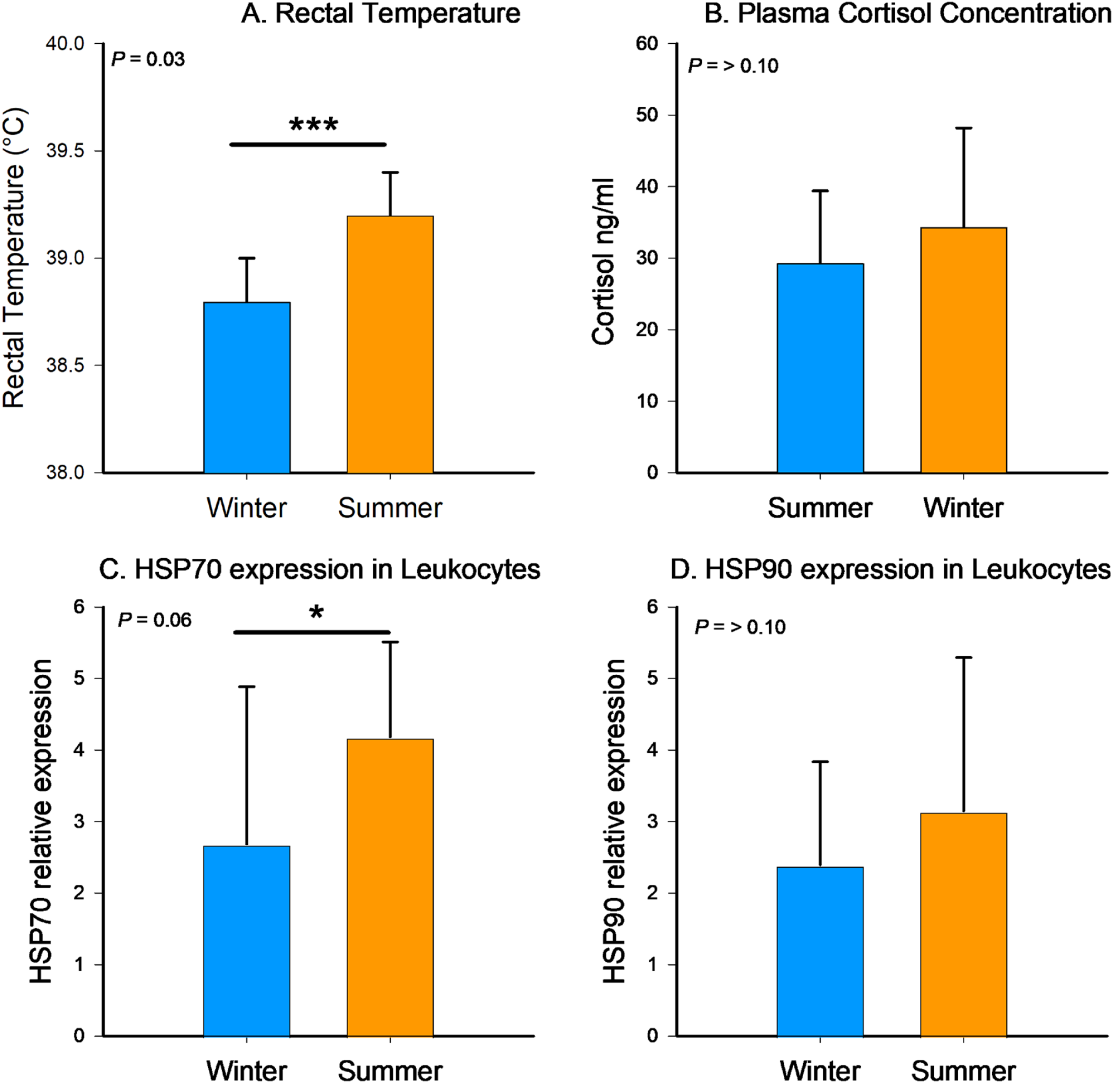
Mean ± SEM of rectal temperature (**A**), plasma cortisol concentration (**B**), and *HSP70* (**C**) and *HSP90* (**D**) transcript abundance from beef cows during the summer and winter (*n* = 11). *** Denotates statistical differences when *P* ≤ 0.05 and * denotates statistical tendencies when *P* ≥ 0.05 still *P* < 0.1

Average rectal temperatures were higher (*P* = 0.03) in the summer (39.2 ± 0.2 °C) than in the winter (38.8 ± 0.2 °C; Fig. 3A). However, plasma cortisol concentrations did not differ when comparing collections from the summer (29.4 ± 3.0 ng/mL) and winter (34.4 ± 4.2 ng/mL) seasons (Fig. 3B).

#### *HSP70* and *HSP90* transcript abundance in leukocytes

Quantitative analysis of *HSP70* and *HSP90* in leukocytes isolated from cows during the summer and winter seasons revealed that *HSP70* tended to have a higher abundance during the summer season, with no difference in the expression of *HSP90* (Fig. 3C and D).

### Follicular observations and oocyte recovery

No significant difference in the total number of follicles (summer 23.9 ± 3.9 vs. winter 18.9 ± 4.8) and the total number of oocytes recovered (summer 13.0 ± 3.6 vs. winter 9.0 ± 1.6) between seasonal collections was observed (Table 2). However, oocyte quality differed when comparing the seasons. In summer, both the number (summer 1.09 ± 0.5 vs. winter 6.4 ± 1.7; P = < 0.01) and the proportion (summer 7.1% ± 2.5 vs. winter 57.8% ± 12.0; P = < 0.01) of grade 1 oocytes decreased, while the number (summer 4.1 ± 1.1 vs. winter 1.2 ± 0.7; *P* = 0.09) and the proportion (summer 30.5% ± 5.5 vs. winter 12.3% ± 5.2; *P* = 0.04) of grade 4 oocytes increased or tended to increased. The number of grade 2 and grade 3 oocytes did not differ significantly between seasonal collections, however, the proportion of grade 2 (summer 26.3% ± 8.4 vs. winter 11.4% ± 5.6; *P* = 0.05) and grade 3 (summer 36.1% ± 5.4 vs. winter 8.3% ± 8.3; P = < 0.01) oocytes was higher from collections during the summer compared to the winter (Table 2).

**Table 2:** Number of follicles visualized at OPU and of recovered oocytes and their quality in summer and winter

Variables (Mean ± SEM)	SUM	WIN	*P* value
	*Numbers*		
Total follicles	23.9 ± 3.9	18.9 ± 4.	0.57
Total oocytes	13.5 ± 0.2	9.3 ± 1.6	0.36
Grade 1	1.09 ± 0.5	6.4 ± 1.7	< 0.01
Grade 2	3.2 ± 1.3	1.4 ± 0.7	0.13
Grade 3	5.1 ± 1.5	0.6 ± 0.6	0.64
Grade 4	4.1 ± 1.1	1.2 ± 0.7	0.09
	*Percentage*		
Grade 1	7.1 ± 2.5	57.8 ± 12.0	< 0.01
Grade 2	26.3 ± 8.4	11.4 ± 5.6	0.05
Grade 3	36.1 ± 5.4	8.3 ± 8.3	< 0.01
Grade 4	30.5 ± 5.5	12.3 ± 5.2	0.04

### Oocyte transcriptome analysis

**Fig. 4:**
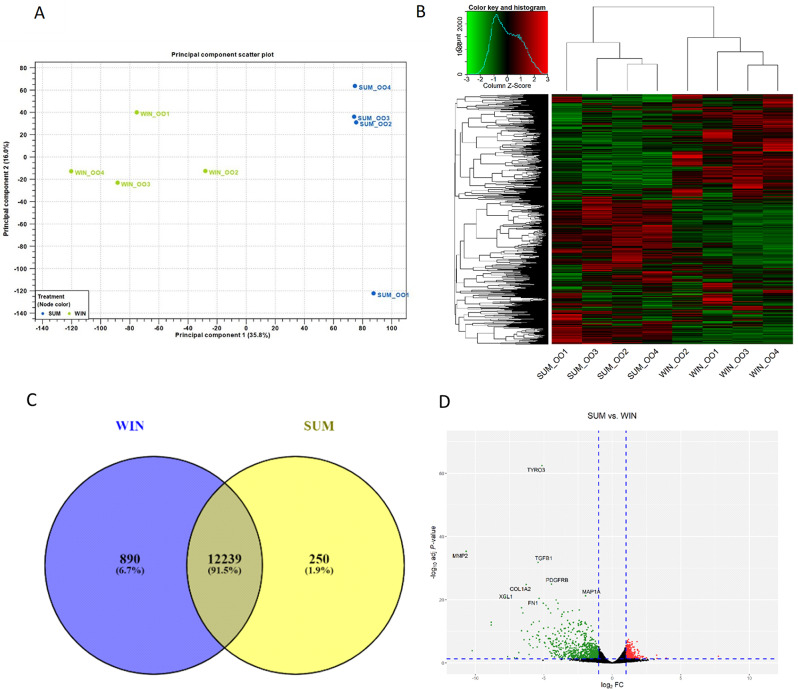
Transcriptomic analyses and differentially expressed genes (DEGs) during summer and winter of oocytes in beef cows. Principal component analysis (PCA; **A**) and hierarchical heatmap (**B**) indicating the expression of genes in oocyte’s during summer and winter. Venn diagram (**C**) showing exclusively and mutually expressed genes between summer and winter and Volcano plot (**D**) indicating the differentially expressed genes from summer compared to the winter [Individual red and green dots represent the number of up- and downregulated genes, respectively (FC ≥ 2; FDR p-value ≤ 0.05)]. Summer = SUM (*n* = 4) and Winter = WIN (*n* = 4)

After quality control, data from 8 libraries (*n* = 4 for summer and *n* = 4 for winter) were used for the bioinformatic analyses. The eight cDNA libraries had an approximate average of 53 million clean reads per library, with an average of 98.4% of the reads mapped to the bovine reference genome. The principal component analysis (PCA) in Fig. 4A and hierarchical heatmap in Fig. 4B displayed clear separation and distribution based on the expression of genes for each group. A total of 12,489 and 13,129 genes were expressed (zFPKM > -3 in the four replicates) in the summer and winter groups, respectively with 12,239 genes being mutually expressed during both seasons. Alternatively, 250 genes were expressed exclusively in the summer, while 890 genes were expressed exclusively in the winter (Fig. 4C). Among the top 20 expressed genes, 16 were detected in both groups, including Ubiquitin B *(UBB)*, Serglycin *(SRGN)*, Inhibin Subunit Alpha *(INHA)*, Cytochrome C Oxidase I *(COX1)*, Cytochrome C Oxidase III *(COX3)*, and Heat Shock Protein Family B (Small) Member 1 *(HSPB1)* (Table 3).

**Table 3:** The top 20 most abundant expressed genes in the oocytes from the summer (SUM) and the winter (WIN), represented as the mean of TPM. Common genes in SUM and WIN are bolded

Gene symbol	SUM, TPM	Gene symbol	WIN, TPM
***UBB***	8675.86	***SRGN***	7458.34
***SRGN***	6404.08	***INHA***	6818.34
***HSPB1***	4652.46	***COX3***	6490.96
***VIM***	4635.51	***VIM***	6143.22
***ACTG1***	4398.71	***UBB***	5878
***COX3***	4098.08	***ACTG1***	5643.2
***RPLP0***	4082.57	***COX1***	5413.63
***RPS15***	4031.76	***HSPB1***	5185.75
***RPLP1***	3724.83	***RPS15***	4297.18
***ACTB***	3655.07	***RPLP0***	4251.25
***RPS5***	3647.72	*ATP6*	4164.03
***ACCSL***	3629.19	***ACTB***	4132.6
***COX1***	3460.1	***RPLP1***	4119.52
***INHA***	3214.14	***TPT1***	4013.41
***GAPDH***	3181.01	***ENO1***	3948.27
***TPT1***	3156.35	*ND3*	3906.64
***RPL7A***	3153.91	***GAPDH***	3719.85
***ENO1***	2934.14	*SERPINE2*	3687.72
***RPL18A***	2807.29	***RPS5***	3619.45
***ZP3***	2770.11	*ND2*	3455.46

TPM-Transcript per million

Differential expression analysis indicated a total of 1,386 genes differentially expressed between the two groups (FC ≥ 2; FDR = ≤ 0.05; zFPKM>-3) in all biological replicates of the enriched group (Supp Table 2). During the summer, there was an up-regulation of 446 genes and a downregulation of 940 genes compared to the winter (Fig. 4D). Matrix Metallopeptidase 2 (*MMP2) was the* top downregulated gene (FC = -1628.99) and Tumor necrosis factor Receptor Superfamily Member 13B (*TNFRSF13B)* was among the top upregulated genes; (FC = 213.56; Table 4).

**Table 4:** Top 10 up- and downregulated, genes in the oocytes from the summer (SUM) compared to the winter (WIN) groups

Name	FC	FDR
*TNFRSF13B*	213.56	0.008224
*ENSBTAG00000050942*	15.23	0.033098
*GAST*	9.56	0.040492
*ENSBTAG00000030892*	9.41	0.003428
*DRD5*	5.62	0.023822
*PPYR1*	5.18	0.038289
*EFNA3*	4.82	0.009969
*VSX1*	4.57	0.016235
*TLX3*	4.52	0.008296
*ENSBTAG00000018583*	4.20	0.013834
*COL3A1*	-98.37	3.38E-18
*TEAD4*	-113.22	0.000543
*TNFRSF8*	-124.88	0.028745
*TNFSF4*	-141.01	0.023035
*C1QTNF5*	-200.22	0.011001
*XCL1*	-223.48	1.18E-21
*EMP1*	-456.81	1.04E-12
*ANXA8L1*	-457.97	1.34E-13
*TMEM26*	-1198.66	0.000143
*MMP2*	-1628.99	4.78E-36

FC-Fold change, FDR-False discovery rate

**Fig. 5:**
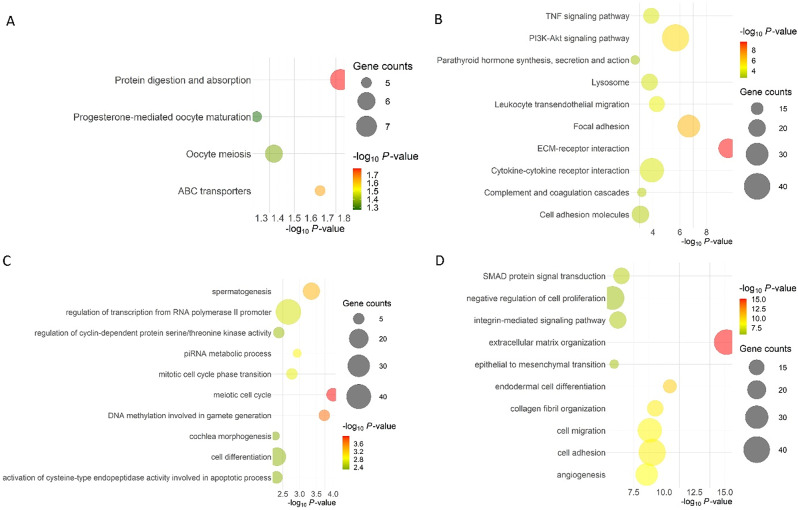
Top 20 enriched pathways (**A**. Upregulated pathways; **B**. Downregulated pathways) and biological processes (BP; **C**. Upregulated BP; **D**. Downregulated BP) in oocytes collected during summer (SUM) and winter (WIN) in beef cows

Gene enrichment analysis of the oocytes identified protein digestion and absorption, ABC transporters, oocyte meiosis, and progesterone-mediated oocyte maturation as the top significant pathways upregulated in summer (Fig. 5A). While ECM receptor interaction, phosphoinositide 3-kinase (*PI3K*)–*AKT* (*PI3K-AKT*) signaling, and focal adhesion pathways were the top-significant pathways downregulated (Fig. 5B). Moreover, the meiotic cycle and DNA methylation involved in gamete generation were the top significant biological processes upregulated in summer and extracellular matrix organization was the top downregulated (Fig. 5C & D).

### Granulosa cells transcriptome analysis

**Fig. 6:**
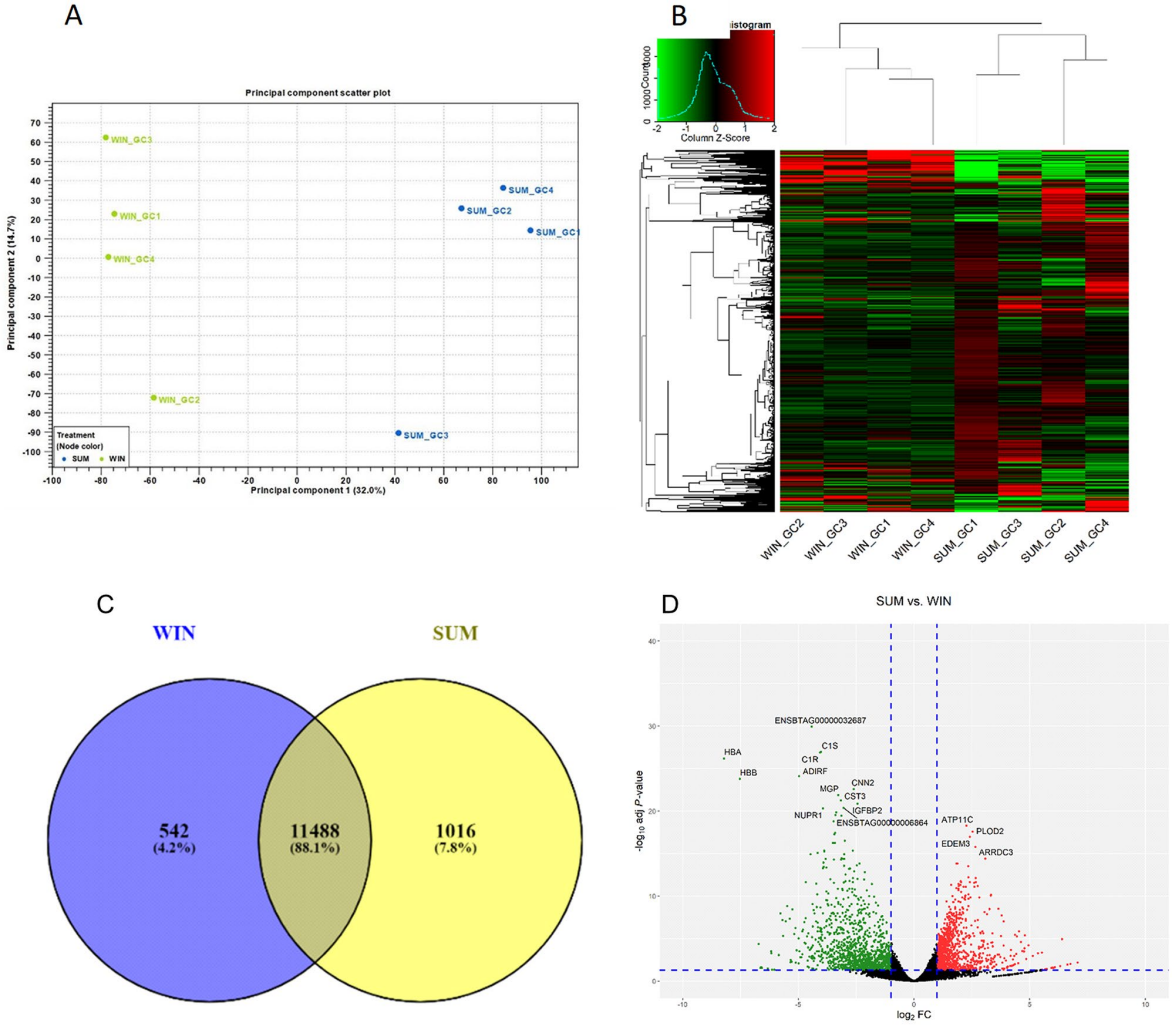
Transcriptomic analyses and differentially expressed genes (DEGs) during summer and winter of granulosa cells in beef cows. Principal component analysis (PCA; **A**) and hierarchical heatmap (**B**) indicating the expression of genes in granulosa cells during summer and winter. Venn diagram (**C**) showing exclusively and mutually expressed genes between summer and winter and Volcano plot (**D**) indicating the differentially expressed genes from summer compared to the winter [Individual red and green dots represent the number of up- and downregulated genes, respectively (FC ≥ 2; FDR p-value ≤ 0.05)]. Summer = SUM (*n* = 4) and Winter = WIN (*n* = 4)

Data from 8 libraries (*n* = 4 for summer and *n* = 4 for winter) were used in the bioinformatic analyses following quality control. The eight cDNA libraries had an approximate average of 42 million clean reads per library, with an average of 98.57% of the reads mapped to the bovine reference genome. The PCA (Fig. 6A) and hierarchical heatmap (Fig. 6B) displayed clear separation and distribution based on the expression of genes for each group. A total of 12,504 and 12,030 genes were expressed (zFPKM > -3 in the four replicates) in the summer and winter groups, respectively, with 11,488 genes being mutually expressed in both groups. Alternatively, 1016 genes were expressed exclusively in the summer, while 542 genes were expressed exclusively in the winter (Fig. 6C). Among the top 20 expressed genes, 16 genes were also mutually represented amongst both groups (Table 5), including *COX1-3*,* ND1-6*,* ATP6*, and *ATP8* with *COX1* and *COX3* represented as the top 2 genes in both seasons.

**Table 5:** The top 20 most abundantly expressed genes in the granulosa cells of the summer (SUM) and the winter (WIN) groups, represented as the mean of TPM. Common genes in SUM and WIN are bolded

Gene symbol	SUM, Avg TPM	Gene symbol	WIN, Avg TPM
***COX1***	193,132	***COX1***	212,379
***COX3***	125,826	***COX3***	165,935
***ATP6***	72772.2	***ND3***	126,366
***ND1***	53169.8	***ATP6***	83449.6
***CYTB***	51791.2	***COX2***	72552.4
***COX2***	48233.7	***ND1***	59318.4
***ND3***	45521.3	***CYTB***	54185.6
***ND4***	43397.3	***ND4***	39361.5
***ND2***	32595.8	***ND2***	24648.4
***ND5***	22146.8	***ND6***	19,144
***ND6***	14126.5	***ND5***	18328.2
***INHA***	10505.2	***ND4L***	9892.43
***SRGN***	5114.24	***ENSBTAG00000043570***	9711.51
***ENSBTAG00000043570***	5063.53	*ENSBTAG00000043567*	5481.22
***ND4L***	4927.43	***ATP8***	2139.79
***GSTA1***	4899.34	***GSTA1***	1694.48
***SERPINE2***	4193.81	*RPLP1*	1181.72
***ATP8***	3699.33	***INHA***	1064.28
***HSPB1***	3475.47	*RPL18A*	1023.44
***INHBA***	2863.98	*HBA*	868.832

TPM-Transcript per million

**Fig. 7:**
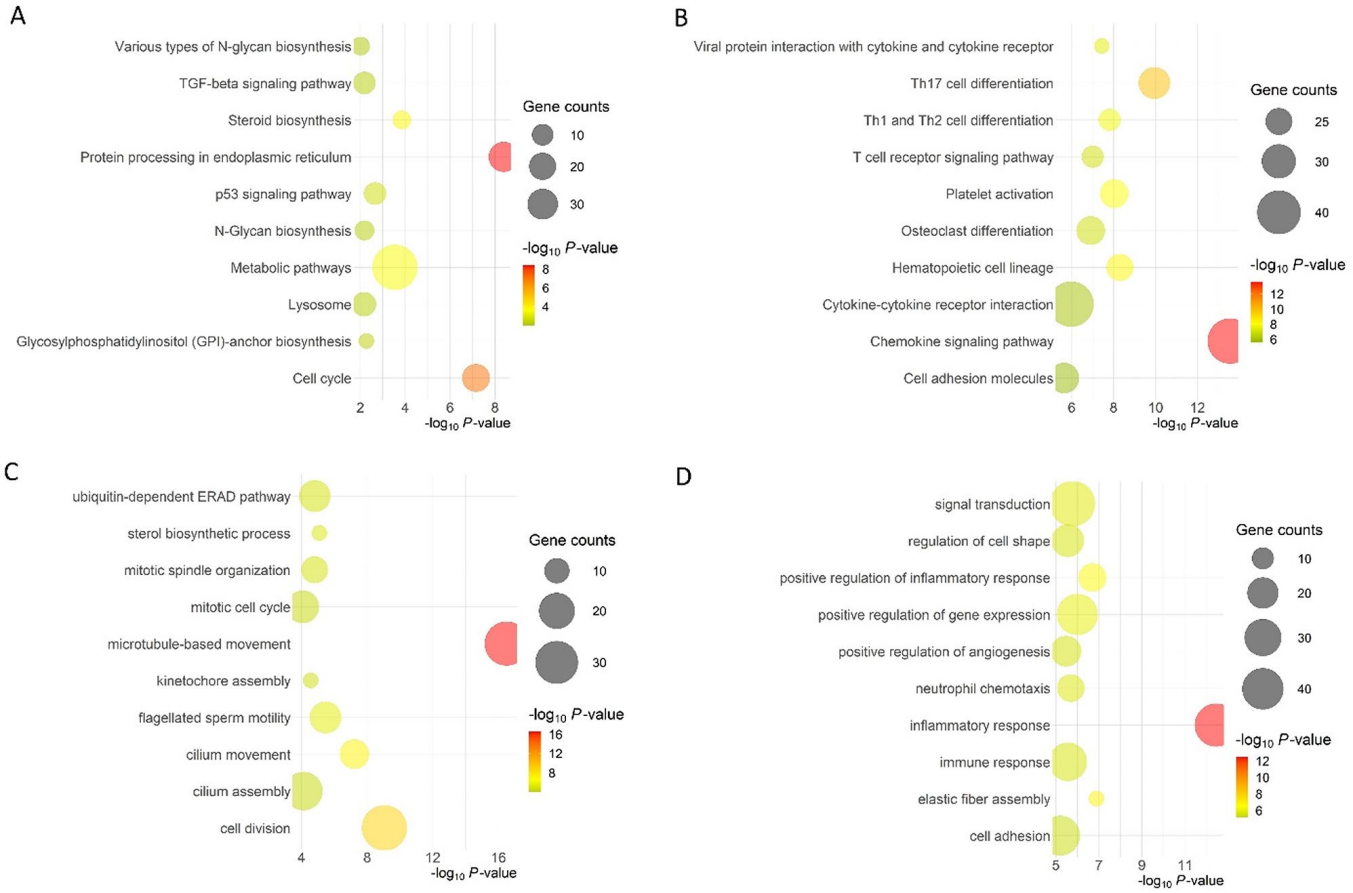
Top 20 enriched pathways (**A**. Upregulated pathways; **B**. Downregulated pathways) and biological processes (BP; **C**. Upregulated BP; **D**. Downregulated BP) in granulosa cells collected during summer (SUM) and winter (WIN) in beef cows

Differential expression analysis indicated a total of 2,209 genes significantly differentially expressed between the two seasons (FC ≥ 2; FDR = ≤ 0.05; zFPKM>-3) in all replicates of the enriched group; Supp Table 3). Comparing the granulosa cells collected during the summer and the winter groups, there was an up-regulation of 1083 genes and a down-regulation of 1126 genes (Fig. 6D). Table 6 represents the top 10 up- and downregulated DEGs. Gene enrichment analysis of DEGs in granulosa cell samples identified protein processing in the endoplasmic reticulum and cell cycle as the top-significant upregulated pathways in summer, while chemokine signaling and Th17 cell differentiation were the top downregulated (Fig. 7A & B). When analyzing biological processes enrichment, microtubule-based movement and cell division were the top upregulated, while the inflammatory response was the top downregulated in summer (Fig. 7C & D).

**Table 6:** Top 10 up- and downregulated genes in the granulosa cells from the summer (SUM) compared to the winter (WIN)

Name	FC	FDR
*ENSBTAG00000050418*	134.88	0.006551
*DNAH10*	108.88	0.009549
*LRRC71*	98.71	0.011828
*DNAH11*	84.21	1.19E-05
*ACCSL*	79.89	0.018649
*DNAH6*	68.15	0.024894
*TMEM151B*	65.44	0.028451
*PLEKHS1*	63.40	0.027665
*ZP2*	61.61	0.029016
*CCDC13*	61.03	0.030493
*NKX2-3*	-70.50	0.046237
*CABLES1*	-71.73	0.000577
*WNT2*	-72.44	0.000320
*CIDEC*	-79.70	0.035243
*ZNF831*	-95.60	0.028134
*GASK1A*	-98.00	0.024834
*NRN1*	-100.16	0.027999
*RNASE1_2*	-104.72	4.51E-05
*HBB*	-185.26	1.68E-24
*HBA*	-296.61	6.38E-27

FC-Fold change, FDR-False discovery rate

### Comparative gene expression analysis of oocyte and granulosa cells

**Fig. 8:**
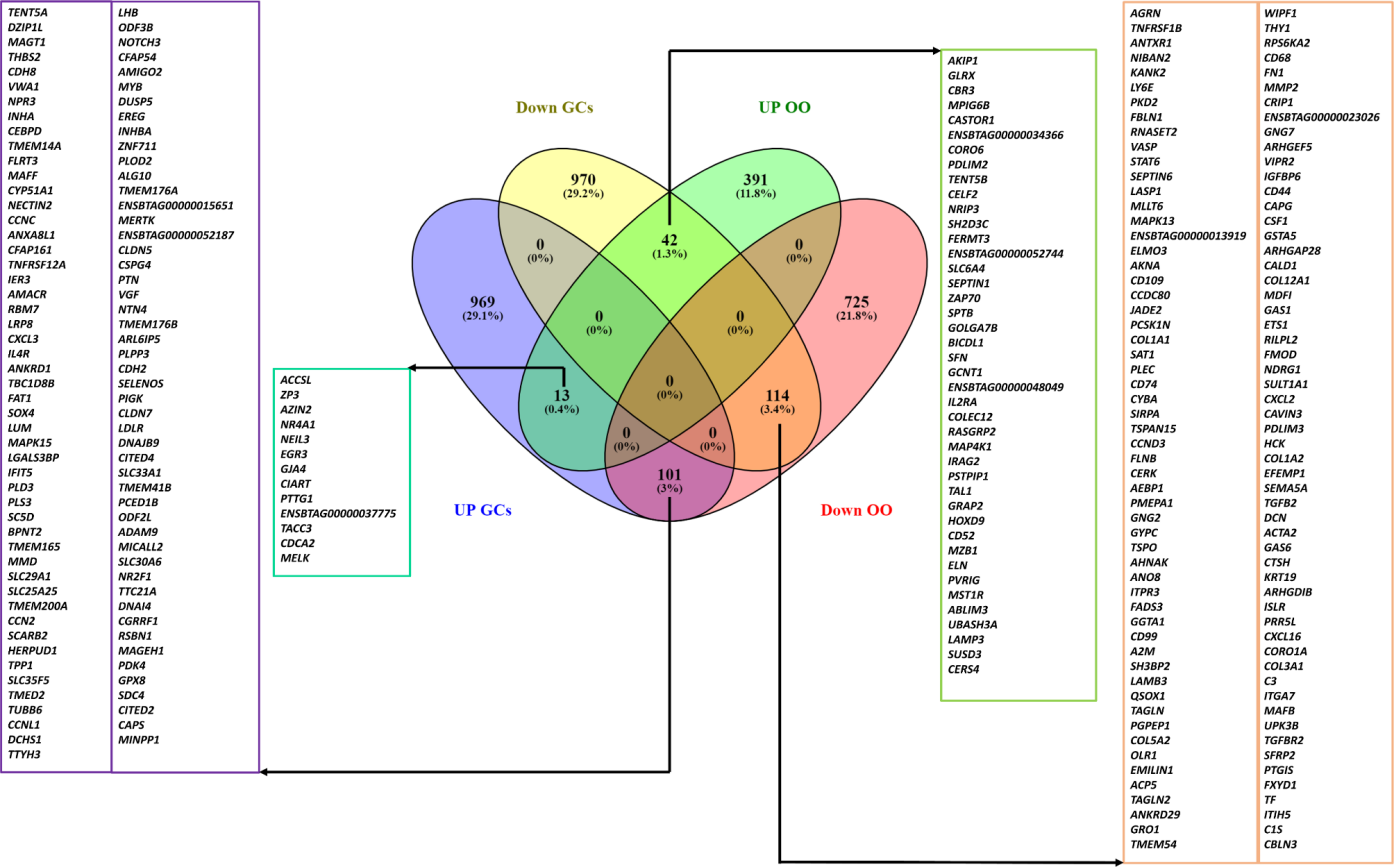
Commonly and exclusively differentially expressed genes from summer compared to winter collected oocytes (OO) and granulosa cells (GCs)

**Fig. 9:**
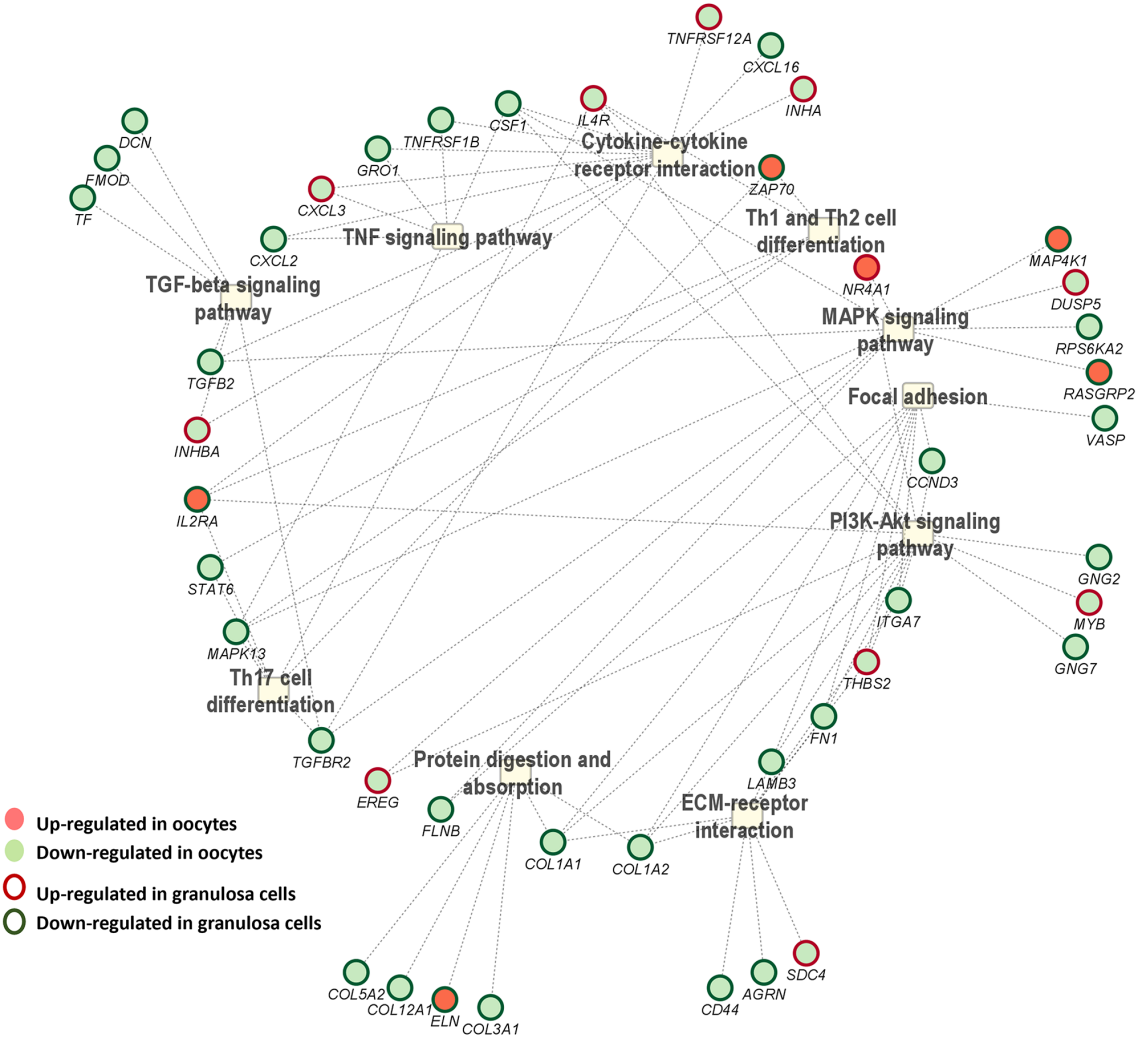
Top pathways and the related commonly DEGs in the oocytes and granulosa cells in summer compared to winter groups

**Fig. 10:**
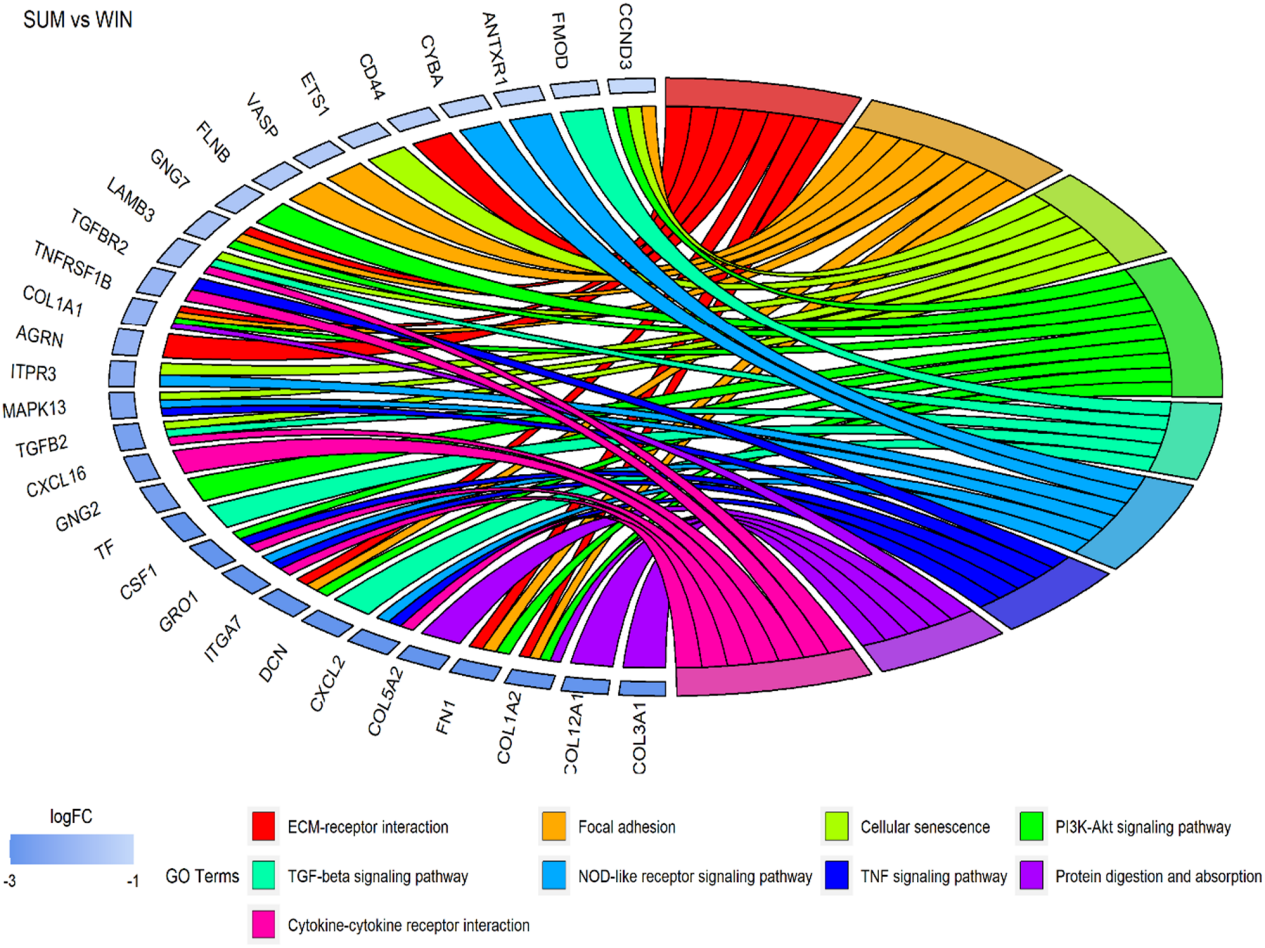
Top pathways and the related DEGs which exhibited a downregulation in oocytes and granulosa cells in summer compared to winter groups

Differential expression analysis revealed a set of 270 genes commonly dysregulated in both oocyte and granulosa cell datasets (Fig. 8). Within this set, 127 genes exhibited a similar expression pattern in which 13 were upregulated and 114 were downregulated in both oocytes and granulosa cells during summer compared to winter conditions. Conversely, 101 genes were upregulated in granulosa cells and downregulated in oocytes while 42 genes showed the opposite pattern, being upregulated in oocytes, and downregulated in granulosa cells (Fig. 8). Pathway analysis of these 270 common DEGs identified several pathways including transforming growth factor-β (*TGF-beta*), TNF, mitogen-activated protein kinase (*MAPK*), and *PI3K-Akt* signaling pathways, as well as cytokine-cytokine receptor interaction, *Th1* and *Th2* cell differentiation, focal adhesion, ECM-receptor interaction, and protein digestion and absorption pathways as the top affected pathways. The interaction network of these pathways and their corresponding DEGs is presented in Fig. 9. Most of these pathways involved genes that were mutually downregulated in both datasets, as depicted in Fig. 10.

## Discussion

Our study aimed to identify differences in the transcriptome of oocytes and granulosa cells collected from beef cows during the summer and winter months. Throughout our study, the THI during the summer period continually deviated from normal levels, likely indicating that the animals within the study were exposed to environmental heat stress as evidenced by their increased rectal temperatures. In *Bos tauru*s beef cattle, a THI over 75 signifies a level of heat stress [22], which was replicated in the present study with the average summer THI being 79.2. While THI has been commonly used as an indicator of heat stress in livestock [23, 24], it does come with certain limitations given that other factors, such as solar radiation and wind speed, are not included in its calculation [25, 26]. In our study, plasma cortisol concentrations did not differ between collections during the summer and winter. Cortisol is a steroid hormone regulated by the hypothalamic-pituitary-adrenal axis [27], in which previous studies in dairy cattle [28–30] have found a significant increase in plasma cortisol concentrations in animals exposed to heat stress. Such discrepancies in cortisol results might be due to the fact that our observations included chronic (long-term) rather than acute (short-term) exposure to levels of heat stress. Additionally, in our study the transcript abundance of *HSP70* in leukocytes tended to increase in the summer (*P* = 0.06), while levels of *HSP90* remained stable during both seasons. In peripheral blood mononuclear cells of beef calves, Kim et al. [31] found that *HSP70* expression was higher during conditions of heat stress, while the expression of *HSP90* showed no differences, concluding that *HSP70* exhibits a more sensitive mechanism than *HSP90*, aiding in the adaptation to heat stress. A major function of HSPs are via their role as indicators of thermotolerance [32], defined as an animal’s ability to balance heat production/dissipation under ambient temperatures above the thermoneutral zone [33]. Therefore, the combination of increased rectal temperatures and the tendency of increased *HSP70* expression in leukocytes during the summer, may indicate that our experimental animals have experienced heat stress prior to the summer OPU collections.

Heat stress affects oocyte quality and subsequently oocyte competence [34], nuclear maturation rate [35], fertilization rate [35], and blastocyst production in vitro [34]. Similarly, in the present study while a higher proportion of grade 1 oocytes was collected during winter, the proportion of grade 4 oocytes was higher in summer collections. In addition, heat stress disturbs the synthesis of steroid hormones (i.e., decrease estradiol although increase progesterone concentration in the follicular fluid) [36] which are directly involved in the mechanisms regulating oocyte maturation. The consistency in these results among different studies should be a concern for the beef industry, where there is low adoption of reproductive biotechnologies that would help improve fertility and pregnancy rates in the summer (such as in vitro embryo production and embryo transfer programs) and where reproductive programs consisting of natural mating with no controlled breeding season (i.e., bulls are allowed to breed cows continuously during the year) are predominantly used [37].

In the current study we showed that oocytes and granulosa cells respond to environmental fluctuations between the summer and winter seasons through the activation or inhibition of genes involved in several pathways associated with ovarian function. We reported here that 1,386 genes were differently expressed in oocytes during the summer and winter. The top upregulated protein-coding genes for oocytes collected during the summer were *TNFRSF13B*, Gastrin (*GAST*), Dopamine Receptor D5 (*DRD5*), Pancreatic Polypeptide Receptor 1 *(PPYR1)*, Ephrin A3 *(EFNA3)*, Visual System Homeobox 1 *(VSX1)*, and T Cell Leukemia Homeobox 3 *(TLX3)*. On the other hand, Collagen Type III Alpha 1 Chain *(COL3A1)*, TEA Domain Transcription Factor 4 *(TEAD4)*, TNF Receptor Superfamily Member 8 (*TNFRSF8*), TNF Superfamily Member 4 (*TNFSF4*), C1q and TNF Related 5 (*C1QTNF5*), X-C Motif Chemokine Ligand 1 (*XCL1*), Epithelial Membrane Protein 1 (*EMP1*), Annexin A8 Like 1 (*ANXA8L1*), Transmembrane Protein 26 (*TMEM26*), and Matrix Metallopeptidase 2 (*MMP2*) were among the top downregulated genes in summer. These dramatic differences in the transcriptome of oocytes collected during the summer and winter seasons indicate both oocytes response to elevated environmental THI and its subsequent impact on the physiology of the oocyte.

Genes involved in regulating cellular proliferation were upregulated in oocytes collected during the summer season. Among the transcripts upregulated, *TNFRSF13B* is a tumor necrosis factor receptor superfamily member previously identified in human and mouse oocytes [38]. *GAST*, which encodes for a peptide hormone key in gastric acid secretion in stomach G cells, was reported to be associated with positive regulation of cell proliferation [39–41]. *EFNA3* is a member of the ephrin (*EPH*) family, a group of molecules known for playing a role in embryonic development, cellular proliferation, migration, and adhesion [42–44]. *DRD5* is expressed in several types of cancers, and treatment with *DRD5* agonists can induce apoptosis and autophagy [45]. These upregulated genes are members of the meiotic cell cycle and cell differentiation pathways, enriched in oocytes collected during the summer season. The upregulation of cellular proliferation markers in immature oocytes from summer compared to winter could be a protective response to the challenging conditions caused by heat stress. However, it can also disrupt the normal developmental process of the oocyte which relies on the balance between proliferation and apoptosis in the COCs, leading to suboptimal maturation or developmental competence [46]. Additionally, the cumulus cells surrounding the oocyte normally undergo a shift from proliferation to differentiation as the oocyte matures. Persistent proliferation markers could suggest incomplete differentiation of these supporting cells [47].

Interestingly, Gendelman and Roth (2012) described an abundance of Proto-Oncogene C-Mos (*C-MOS)*, Growth Differentiation Factor 9 *(GDF9)*, POU Class 5 Homeobox 1 *(POU5F1)*, and Glyceraldehyde-3-Phosphate Dehydrogenase *(GAPDH)* transcripts as higher in MII-stage oocytes collected in the cold season than in those from the hot season [48]. However, in their study, this seasonal variation was not present in GV-stage oocytes [48]. In contrast, our study found that *MOS* and *GDF9* were upregulated in oocytes during the summer season (GV-stage). Also, similar to Ferreira et al. [49], we found an upregulation of Fibroblast Growth Factor 16 (*FGF16)* and *GDF9* in oocytes collected during the summer months compared to those in the winter months. This may signify the fact that oocytes respond to thermal stress in stage dependent manner.

Among the genes downregulated in oocytes collected during the summer season, transmembrane protein 26 (*TMEM26*) and matrix metalloproteinase 2 (*MMP2*) were suppressed by more than a thousand-fold. The latter, i.e., *MMP2*, levels in human follicular fluid were found to be a reliable marker for oocyte maturation in in vitro fertilization and intracytoplasmic sperm injection cycles [50]. Moreover, a recent study by Latorraca et al. [51] identified *MMP2* to be present at all phases of oocyte growth (common to all oocyte size groups, from < 60 μm to > 120 μm). Therefore, the significant reduction of such transcripts in oocytes collected during the summer season implies the negative impact of thermal stress on oocytes’ developmental competence by suppressing transcripts critical for their growth and development. Collectively, double the number of transcripts were found to be downregulated in oocytes collected during the summer compared to their winter counterparts. This fact evidenced the potential impact of elevated environmental temperatures on oocytes competence is via the suppression of the expression of transcripts important for their growth and maturation. This has also been evidenced in the type of pathways in which the downregulated transcripts participate (Fig. 5B). A significant proportion of downregulated transcripts were found to be involved in *PI3K-Akt* signaling and focal adhesion pathways. The *PI3K-Akt* signaling pathway is reported to be a key regulator of many cellular processes associated with cell proliferation, survival, growth, cytoskeletal rearrangement, and metabolism [52], which makes the pathway to be a potential predictor for the developmental competence of oocytes and successful embryo implantation [53]. Similarly, studies in bovine oocytes revealed that follicular granulosa cells’ expression of the *PI3K-Akt* signaling pathway to be correlated with the developmental competence of oocytes after parthenogenetic activation [54]. Therefore, our results demonstrate that one of the mechanisms by which environmental thermal stress impacts oocyte competence is via the suppression of transcripts involved in various key pathways regulating follicular development, oocyte maturation, and further embryonic developmental potential.

Granulosa cells are known to mediate oocyte developmental process via an active bidirectional communication with the oocyte [55, 56]. Therefore, any significant negative impact of environmental seasonal changes on granulosa cell physiology would have a direct impact on ovarian function and oocyte developmental processes. Transcriptome analysis of granulosa cells form summer and winter seasons revealed that among the 2,209 differentially expressed genes, several members of the Heat Shock proteins family (Heat Shock Protein Family A *(Hsp70*) Member 5 (*HSPA5*), Heat Shock Protein 90 Beta Family Member 1 (*HSP90B1*), DnaJ Heat Shock Protein Family (*Hsp40*) Member B9 (*DNAJB9*), B11 (*DNAJB11*), B14 (*DNAJB14*), B13 (*DNAJB13)*, and C5 (*DNAJC25*) were found to be upregulated in granulosa cells during the summer season as a response to higher environmental temperatures. These genes have been previously described as components of the Heat Shock Protein cascade in response to heat stress [57, 58], and have been shown to be elevated in granulosa cells after in vitro exposure to acute heat stress [59, 60]. In addition, here we report that several members of the axonemal dynein complex members including Dynein Axonemal Heavy Chains 1 (*DNAH1*), 5 (*DNAH5*), 6 (*DNAH6*), 7 (*DNAH7*), 9 (*DNAH9*), 10 (*DNAH10*), 11 (*DNAH11*), and 12 (*DNAH12*) to be upregulated in the summer months. Moreover, *DNAH6*,* DNAH10*, and *DNAH11* were in the top 10 upregulated genes in the granulosa cells during summer. Dyneins are a family of cytoskeletal motor proteins that form the microtubules in cells. Dyneins have been localized in follicles (granulosa cells and oocytes) [61, 62], and in mice, the inhibition of dynein proteins significantly increased the number of growing follicles [63].

Other class of transcripts related to Zona Pellucida Glycoprotein 2 (*ZP2*), a critical component of the Zona Pellucida, was in the top 10 upregulated genes in the granulosa cells during summer. Similar studies have also previously indicated that heat stress impacts the ZP function and therefore disrupts the anti-polyspermy system of the oocyte, which resulted in increasing polyspermy [64] and increased number of pores and a more significant percentage of oocytes with amorphous ZPs [65] as a result of thermal stress. Collectively, our data and also from others suggest that in vivo, granulosa cells are also susceptible to seasonal heat stress provoking changes in cell transcriptome dynamics, which could be possibly related to the decrease in oocyte quality in the summer.

As observed in oocytes, granulosa cells collected during summer showed upregulation of genes involved in protein processing in endoplasmic reticulum, while downregulated genes are involved in chemokine signaling pathways. Accumulation of unfolded or misfolded proteins in the endoplasmic reticulum due to various environmental stressors induced the onset of ER stress, which ultimately leads to cellular apoptosis [66]. One of the coordinated responses to accumulated unfolded protein in the ER is called unfolded protein response (UPR). Therefore, the upregulation of genes associated with protein processing in ER in the current study revealed that increased protein processing in the ER is one of the mechanisms in which granulosa cells respond to elevated environmental temperature.

Integration of the oocyte and granulosa cells transcriptome data revealed differential regulation of 127 genes in common with a similar direction of expression in both oocytes and granulosa cells in response to environmental temperature. The majority of the transcripts (114 genes) were found to be suppressed in both oocytes and granulosa cells collected during the summer season compared to the winter. Pathway analysis for these commonly suppressed transcripts due to thermal stress in oocytes and granulosa cells revealed their enrichment in several pathways associated with ovarian function, follicle and oocyte development (Fig. 10). The involvement of the *TGF-beta* and *PI3K-Akt* pathways suggests alterations in cell growth, apoptosis, and metabolic processes, which are critical for follicular maturation and oocyte quality [53, 67]. Additionally, changes in focal adhesion and ECM-receptor interactions indicate potential disruptions in oocyte-follicle communication and intracellular signaling [68] due to the elevated summer temperature. Investigating the functional consequences of these gene expression changes on further embryonic development in vivo could provide deeper insights into how environmental stressors affect reproductive efficiency. This is the first evidence indicating how follicular cells (granulosa cells) and gametes (oocytes) are affected by or respond to environmental elevated temperature in the ovarian follicle.

## Conclusion

Our results indicate that cows exposed to seasonal fluctuations of heat stress dramatically alter their granulosa cells and oocytes transcriptome, which may in part be responsible for negatively impacting follicular physiology. Specified genes and their associated pathways and biological processes pose an essential function in bovine granulosa cells and oocytes’ response to heat stress, potentially serving as mechanisms for future targets to mitigate the impact of heat stress within the intrafollicular environment.

## Electronic supplementary material

Below is the link to the electronic supplementary material.

Supplementary Material 1

## Data Availability

The raw FASTQ files and processed CSV files of the RNA sequencing data have been deposited in the NCBIs Gene Expression Omnibus (GEO) repository with the accession numbers GSE235170 and GSE235171.

## Declarations

### Footnotes

N/A.

### Disclaimer

We declare that in part some of the results described in this manuscript were adapted from Kamryn Joyce’s Master’s Thesis.

## Abbreviations

ANXA8L1: Annexin A8 Like 1
ATP6: Mitochondrially Encoded ATP Synthase Membrane Subunit 6
ATP8: Mitochondrially encoded ATP synthase membrane subunit 8
BP: Biological processes
C1QTNF5: C1q and TNF Related 5
C-MOS: Proto-Oncogene C-Mos
COCs: Cumulus-oocyte-complexes
COL3A1: Collagen Type III Alpha 1 Chain
COX1: Cytochrome C Oxidase I
COX3: Cytochrome C Oxidase III
CV: Coefficient of variation
DEGs: Differentially expressed genes
DNAH 1, 1, 5, 6, 7, 9, 10, 11, and 12: Dynein Axonemal Heavy Chains 1, 5, 6, 7, 9, 10, 11, and 12
DNAJB 11, 13, 14, and 90: DnaJ Heat Shock Protein Family (Hsp40) Members B11 B13, B14, and B90
DNAJC25: DnaJ Heat Shock Protein Family (Hsp40) Member C25
DRD5: Dopamine Receptor D5
ECM: Extracellular Matrix
EFNA3: Ephrin A3
EMP1: Epithelial Membrane Protein 1
EVs: Extracellular vesicles
FAWN: Florida Automated Weather Network
FDR: False discovery rate
FGF16: Fibroblast Growth Factor 16
GAPDH: Glyceraldehyde-3-Phosphate Dehydrogenase
GAST: Gastrin
GDF9: Growth Differentiation Factor 9
GEO: NCBI’s Gene Expression Omnibus
GLM: Generalized Linear Model
GV: Germinal vesicle
Hsp40: DnaJ Heat Shock Protein Family
HSP70: Heat shock protein 70
HSP90: Heat shock protein 90
HSP90B1: Heat Shock Protein 90 Beta Family Member 1
HSPA5: Heat Shock Protein Family A (Hsp70) Member 5
HSPB1: Heat Shock Protein Family B (Small) Member 1
INHA: Inhibin Subunit Alpha
MAPK: Mitogen-activated protein kinase
MMP2: Matrix Metallopeptidase 2
ND1-6: NADH dehydrogenase subunits 1–6
OPU: Ovum Pick up
PCA: Principal component analysis
PI3K-AKT: Phosphoinositide 3-kinase (PI3K)–AKT pathway
POU5F1: POU Class 5 Homeobox 1
PPYR1: Pancreatic Polypeptide Receptor 1
qPCR: Quantitative real-time PCR
SRGN: Serglycin
TEAD4: TEA Domain Transcription Factor 4
TGF-beta: Transforming growth factor-β
THI: Temperature humidity index
TLX3: T Cell Leukemia Homeobox 3
TMEM26: Transmembrane Protein 26
TMM normalization: Trimmed mean of the M-values normalization method
TNF: Tumor necrosis factor
TNFRSF13B: TNF Receptor Superfamily Member 13B
TNFRSF8: TNF Receptor Superfamily Member 8
TNFSF4: TNF Superfamily Member 4
TPM: Transcripts per million
UBB: Ubiquitin B
UPR: Unformed protein response
VSX1: Visual System Homeobox 1
XCL1: X-C Motif Chemokine Ligand 1
ZP2: Zona Pellucida Glycoprotein 2
